# Lung Cancer Incidence in Counties at Low and High Risk of Radon Exposure: A Population-Based SEER Analysis (1975–2022)

**DOI:** 10.1101/2025.08.07.25333180

**Published:** 2025-09-02

**Authors:** Nicholas Maxfield, Jason Semprini

**Affiliations:** Des Moines University

## Abstract

**Background:**

Lung cancer is the leading cause of cancer-related mortality in the United States. While smoking cigarettes remains the dominant risk factor, declining smoking rates have drawn attention to Radon. Despite the known risks from individual exposure to radon, less is known about long-term trends in Radon-related lung cancer at the population-level.

**Methods:**

Using SEER-8 cancer registry data from four states, we compared lung cancer incidence between counties classified as low-risk and high-risk (>4.0 pCi/L) for radon exposure. Constructing a random effects Generalized Least Squares regression, which adjusted for county-level smoking prevalence, we quantified the excess lung cancer rate attributable to radon risk across decade, sex, and histologic subtype.

**Results:**

Compared to low-risk counties, counties at high risk of radon exposure had a significantly higher overall lung cancer incidence: 13.5+ cases per 100,000 person-years (95% CI: 10.0, 17.1). This gap between low and high-risk counties was largest for Adenocarcinoma and small cell carcinoma, and larger in males than females. Only in females did we observe the gap in lung cancer incidence between low- and high-risk counties grow overtime.

**Conclusions:**

This study illustrates how county-level radon data can be leveraged to enhance cancer surveillance and guide prevention and control strategies.

## Introduction

Lung cancer kills nearly 125,000 Americans annually[1]. Cigarette smoking contributes the most to lung cancer incidence[2]. However, smoking rates reached historic lows and continue to decline[3,4]. As a result, lung cancer incidence and mortality have declined dramatically[5]. Predictably, given that males smoke more frequently and at higher intensity than females, the observed 30-year decline in lung cancer for males (−38%) was remarkably greater than the stagnate trends in females (−1%)[5–8]. As smoking rates continue declining through the next decade, public health systems have the opportunity to prioritize secondary risk factors in the fight against lung cancer, which, despite 30-year declines, remains the leading cause of cancer mortality in the United States[1,9].

Second only to smoking, and first among non-smokers, the leading risk factor for lung cancer is Radon[10]. Radon has historically accounted for 10–15% of all lung cancers, but unlike the well known risk from smoking, the general public as well as clinicians appear unaware of the risks from Radon[10–12]. A naturally occurring radioactive gas formed by decaying uranium and radium, Radon seeps into buildings from the environment (i.e., soil, rocks, ground)[10]. Radon itself is harmless to humans, but its decay emits radioactive particles capable of attaching to dust, entering our lungs, and damaging our DNA[10,13–15].

In 1988, the International Agency for Research on Cancer classified Radon as carcinogenic to humans[16]. Around this same time in the United States, the Environmental Protection Agency (EPA) ramped up efforts to measure Radon across the country with the goal of understanding Radon’s impact on health[17]. In 1992, an EPA report identified a dose-response relationship between exposure to radon and lung cancer risk, as well as a synergistic relationship in people with a history of smoking[18]. Radon’s negative impact on health, apparently specific only to lung cancer, has since been observed consistently in various settings and communities[19–24].

### Objective

For nearly forty years, the scientific community has known that exposure to higher levels of Radon poses a risk to individuals. Yet, in our fight against lung cancer, two critical evidence gaps remain. First, most of the research since the 1988 classification has focused on individual-level risk of developing lung cancer after exposure to Radon as opposed to population-level surveillance. While individual-level research helps us understand and quantify the risk, population-level surveillance can directly inform public health system strategies. The one population-based study which analyzed Radon and lung cancer, included but a single state and just twenty years of data[23]. Second, while we know lung cancer incidence is declining as smoking rates decline, we know very little about changing trends in lung cancer attributable to Radon. One study attempted to investigate this trend, but failed to account for smoking prevalence and relied on broad national Radon level average; both of which appear inappropriate for understanding Radon attributable lung cancer trends in the United States[25].

Our study aimed to address these evidence gaps and examine the relationship between residential radon exposure and lung cancer incidence by sex, histologic subtype, and decade over a nearly 50-year time period. We hoped to inform public health strategies balancing smoking cessation and radon mitigation efforts in high-risk Radon areas.

## Methods

### Data and Measures

We analyzed data from the Surveillance, Epidemiology and End-Results (SEER-8) program (1975–2022)[26]. SEER-8 is a population-based cancer dataset of the original eight SEER cancer registries. For this study, we restricted our analysis to full state registries in the contiguous United States (Connecticut, Iowa, New Mexico, Utah). All cancer incidence rates were adjusted for age and analyzed at the county level. We further restricted our analysis to lung cancers (Lung and Bronchus). All rates were reported as cases per 100,000 person-years. In addition to reporting overall rates, we stratified the rates by sex (male, female), histologic subtype (Adenocarcinomas ICDO-3: 8140–8389; Squamous cell carcinomas ICD-O-3: 8050; Small cell carcinomas ICD-O-3: 8043/3, 8044/3, 8045/3; and other), and decade of diagnosis (1975–1984, 1985–1994, 1995–2004, 2005–2014, 2015–2022). All counties were then classified into two groups, based on the EPA’s Map of Radon Zones[27]. This map categorizes U.S. counties based on radon measurements, geology, radioactivity, soil, and housing data. The EPA has established Radon levels above 4 pCi/L as high risk for developing lung cancer[28]. Therefore, we classified counties as “high-risk” if they exceeded the EPA’s mitigation threshold of 4.0. All other counties below 4.0 pCi/L were classified as “low-risk”.

### Statistical Analysis

To quantify the difference in lung cancer incidence rates between low and high-risk counties, we constructed a series of Random Effects Generalized Least Squares regression models[29–31]. Each model included a year fixed-effect, which adjusts for unobserved temporal trends in lung cancer incidence consistent across all counties. Additionally, each model included a county-level Random Effect, which adjusts for time-invariant differences between counties. Each model also adjusted for time-invariant county-level smoking differences [32]. For unbiased estimation, we assumed that the Random Effects component was uncorrelated with the other county level regressors. For inference, we estimated standard errors robust to autocorrelation and heteroskedasticity[33,34].

Equation 1 specifies the model, where Y_it_ is the lung cancer incidence rate for county = i, in ten-year period = t. The county-specific Random Effect is u_i_ and the idiosyncratic error term is e^i^. B is the parameter of interest, estimating the independent difference in lung cancer incidence rate between low and high-risk counties.


1) $$Y_{it}=B\;{\text{HIGHRISK}}_{i}+{\text{TENYEAR}}_{t}^{\prime }+{\text{SMOKE}}_{i}+u_{i}+e_{i}$$

In addition to estimating overall differences between low and high-risk counties, we also tested if the difference changed over time. We modified Equation 1 by interacting the HIGHRISK variable with a set of decade (TENYEAR’) dummy variables.


2) $$Y_{it}=B\prime {\text{HIGHRISK}}_{i}*{\text{TENYEAR}}_{t}^{\prime }+{\text{HIGHRISK}}_{i}+{\text{TENYEAR}}_{t}^{\prime }+{\text{SMOKE}}_{i}+u_{i}+e_{i}$$

Now, Equation 2 estimated four B-parameters, each quantifying the decade-specific difference in lung cancer between low and high-risk counties relative to the difference in the reference decade (1975–1984). Finally, in addition to comparing confidence intervals of the estimates, we constructed a joint Wald test to test if the difference between low and high-risk counties changed over time. We used the Sidak correction method to adjust for multiple hypothesis tests (4 lung cancer subtypes × 4 decade tests × 3 groups), setting our alpha (a) for determining statistical significance at 1-(1–0.05)^(1/48) = 0.001[35].

## Results

### Summary Statistics

Table 1 shows the summary statistics. In the four states included in the analysis (1975–2022), 277,843 lung cancer cases were diagnosed. This corresponded to an age adjusted incidence rate of 56.2 per 100,000 person years. 69% of the cases were diagnosed in counties with high-risk levels of Radon. The incidence rate in low-risk counties was 45.5 cases per 100,000 person-years and the incidence rate in high-risk counties was 60.0 cases per 100,000 person-years. Lung cancer incidence was highest in the states of Connecticut and Iowa, the decades of 1985–1994 and 1995–2004, Adenocarcinoma subtypes, and males.

### Overall Differences in Lung Cancer Incidence

**Figure 1:**
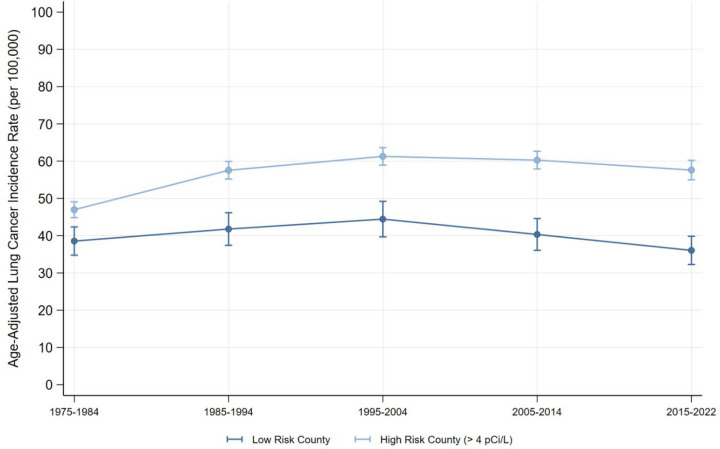
Lung Cancer Incidence (all subtypes) by Radon Exposure Risk Figure 1 visualizes the age-adjusted incidence of lung cancer in low and high-risk Radon counties. Source: SEER-8.

**Figure 2:**
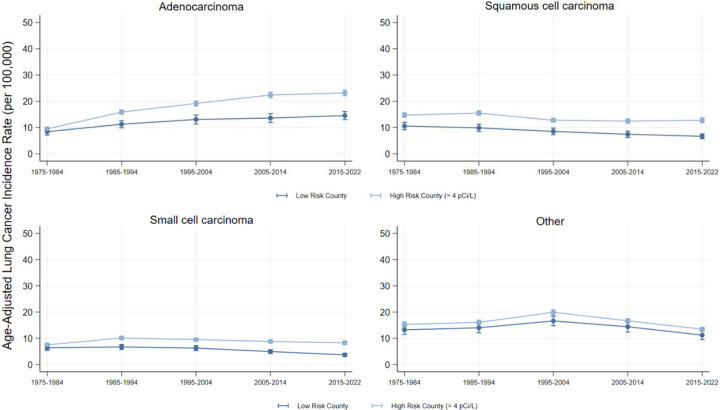
Lung Cancer Incidence by Radon Exposure Risk and Histologic Subtype Figure 2 visualizes the age-adjusted incidence of lung cancer in low and high-risk Radon counties, by histologic subtype. Source: SEER-8.

Overall, after adjusting for smoking rate differences and county-level Random Effects, we estimated that high-risk counties were associated with an additional 13.5 lung cancer cases per 100,000 person-years (CI = 10.0, 17.1] (Table 2). Although each decade specific association was larger than the difference in 1975–1984, peaking in 2015–2022 (Est. = 13.1 cases per 100,000 person-years; CI = 9.0, 17.3), the associations did not vary significantly across decades (Wald Test Statistic p = 0.014). Figure 1 shows the decade-by-decade trends in lung cancer incidence. These overall differences were consistent across histological subtype (Table 2). However, the estimated association between high-risk counties and lung cancer incidence only varied across decades for Adenocarcinoma (Wald Test Statistic p <0.001), again peaking in 2015–2022 (Est. = 7.5 cases per 100,000 person-years, CI = 5.6, 9.5). Figure 2 shows the decade-by-decade trends in lung cancer incidence by histological subtype.

### Differences by Sex

#### Females

For females, we estimated that high-risk counties were associated with an additional 6.8 cases per 100,000 personyears (CI = 3.9, 9.6). Compared each of the three other histological subtypes, the association between high-risk counties and lung cancer incidence among females was larger in Adenocarcinomas (Est. = 3.6 cases per 100,000 person-years, CI = 2.4, 4.8). In fact, among females, there was no difference between low and high-risk counties for “other” lung cancer subtypes (Est. = −0.1 cases per 100,000 person-years, CI = −1.3, 0.4).

**Figure 3:**
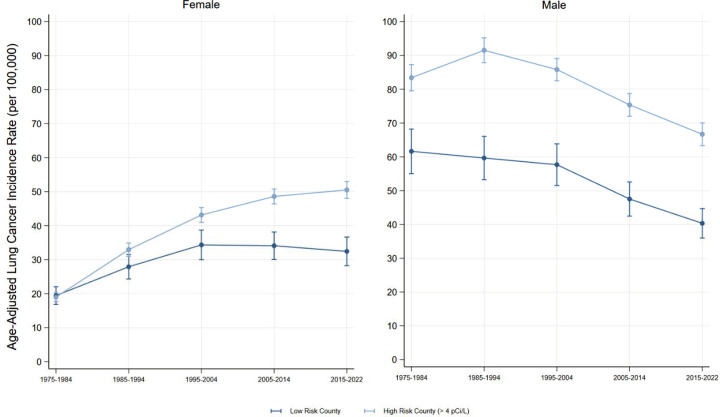
Lung Cancer Incidence (all subtypes) by Radon Exposure Risk and Sex Figure 3 visualizes the female and male age-adjusted incidence of lung cancer in low and high-risk Radon counties. Source: SEER-8.

**Figure 4:**
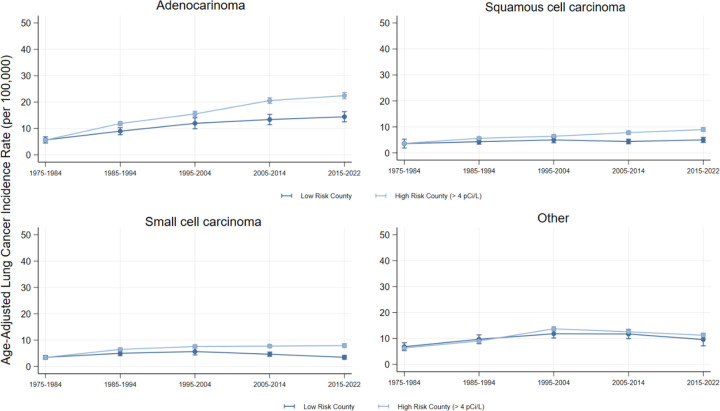
Female Lung Cancer Incidence by Radon Exposure Risk and Histologic Subtype Figure 4 visualizes the female age-adjusted incidence of lung cancer in low and high-risk Radon counties by histologic subtype. Source: SEER-8.

Overall and across the subtypes Adenocarcinoma, Squamous cell, and Small cell, the association between high-risk counties and lung cancer incidence among females grew significantly each decade (Wald Test Statistic p < 0.001). The largest association was for adenocarcinomas in 2015–2022 (Est. = 8.1 cases per 100,000 person-years, CI = 5.6, 10.5). See figures 3–4 for decade-by-decade trends of female lung cancer incidence overall and by subtype.

#### Males

**Figure 5:**
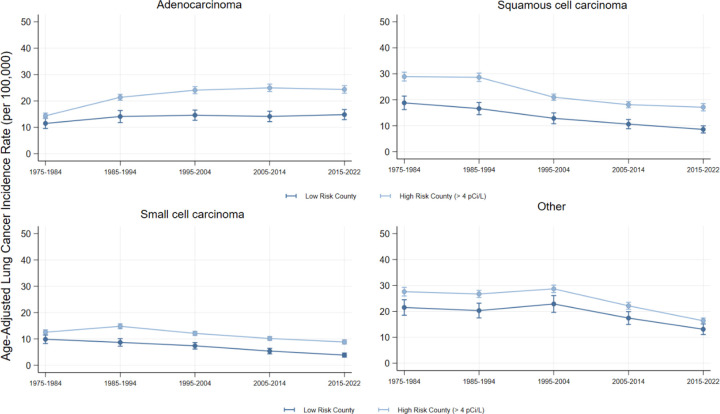
Male Lung Cancer Incidence by Radon Exposure Risk and Histologic Subtype Figure 5 visualizes the male age-adjusted incidence of lung cancer in low and high-risk Radon counties by histologic subtype. Source: SEER-8.

In contrast, Males exhibited larger overall differences between low and high-risk counties, with an estimated association of 23.5 additional cases per 100,000 person-years (CI=18.5, 28.4). Compared to the “other” subtype, the associations were largest for adenocarcinoma and squamous cell carcinoma. Also contrary to the female estimates, the association between high-risk county and lung cancer incidence attenuated over time; where the 2005–2014 and 2015–2022 associations were no longer significantly different than the gap in 1975–1984. In fact, the only subtypes with statistically significant decade-specific coefficients were adenocarcinomas and small cell carcinomas; which again did not vary significantly across decades. See figures 3 and 5 for decade-by-decade trends of male lung cancer incidence overall and by subtype.

## Discussion

Our population-level analysis spanning nearly 50 years highlighted how lung cancer incidence trends varied by county-level of Radon risk. After adjusting for smoking, we observed large differences overall. This result validates the work of Ou, while also affirming that the elevated lung cancer rate in high Radon counties extends beyond the border of a single state[23]. The widening gap over time, however, was concentrated among Adenocarcinomas. While this finding conflicts with previous research (which were notably not population-based, despite claiming to be so, and relied on point-in-time post-diagnosis Radon measures), our finding is supported by the data showing that 50–60% of the lung cancers diagnosed among never-smokers were adenocarcinomas[19,36,37].

Yet, the overall analyses masks sex-specific patterns in lung cancer incidence in high-risk Radon counties. Although the gap between low and high-risk counties was highest in males, that gap appeared to attenuate or stagnate over time. Whereas in females, for each of the three lung cancer subtypes, the gap between low and high-risk counties grew decade after decade. Further, despite only including four small states, the visual trends clearly mirror the overall trends in lung cancer incidence: dramatic declines in males, stagnate trends in females[5]. Since 1985–1994, we found each of the lung cancer types either declined or stagnated for both males and females, except female adenocarcinoma in high-risk Radon counties which has increased dramatically for half a century. Finally, by analyzing the most recent SEER data, we revealed that female lung cancer incidence in high-risk Radon counties now exceeds that of males in low-risk counties.

As smoking rates continue to decline, the proportion of lung cancers attributable to Radon will only grow. These high-risk Radon zones have been identified since 1993 and are unlikely to change, at least in terms of geological risk[27,28]. We cannot change what lies underneath the ground, we can only minimize how Radon emits into our homes, schools, and places of work. Notably, the EPA does not regulate Radon, but only provides education and guidance. States, rather, have been deemed responsible for protecting residents from the harm of Radon. Like most healthcare and public health policies, states have taken a wide range of approaches (testing, reporting, mitigation) in response to the threat of Radon[38,39].

Rather than conclude this study by examining the vast landscape of policy approaches taken, or not taken, we conclude this study by explicitly calling for novel areas of future research. We know Radon poses a significant health risk, potentially driving elevated rates of lung cancer in smokers and never smokers alike. We also know that policymakers have responded differently to this knowledge. What we do not yet know, unfortunately, is what works best at preventing Radon from emitting into homes, schools, and places of work. Neither do we know what works best at reducing downstream lung cancer risk among those with higher propensity of developing lung cancer in the future. To date, no research exists, to our knowledge, that has evaluated the causal impact of state or municipal policies on Radon emissions or lung cancer incidence. While simulation data exists in the U.S., real-world data may be more influential for policymakers[40,41]. This direction of scientific inquiry could involve retrospective analyses of policy changes over time or prospective evaluation (i.e., cluster randomized controlled trials). Such evidence would prove extremely valuable to state Medicaid programs, as well as federal and private health system payers, who pay the eventual cost of caring for patients diagnosed with lung cancer. This evidence would also provide a stronger tool for public health advocates, who currently rely solely on the need to reduce exposure to Radon, by providing concrete cost-effective solutions that advance population health and reduce healthcare expenditures. Although Radon may be a growing driver of lung cancer, especially in never smokers, we would be remise to ignore the synergistic effect of Radon exposure among smokers[42]. The most recent data shows that 14% of American adults still smoke cigarettes, with higher rates among Black or Indigenous populations and people with underlying chronic conditions (i.e., disability, depression)[43]. While public health professionals should be celebrating the historic declines in cigarette smoking, we cannot afford to be complacent. Rather than solely balancing smoking cessation and Radon mitigation, public health systems should explore innovative approaches to combat both threats simultaneously. In fact, previous academic literature has shown the efficacy of leveraging perceived radon risk as a tool to inform the public about Radon and reduce smoking behavior[44–47]. Scaling these direct contact approaches with updated technology platforms across geographies and populations remains paramount[48–50]. States and municipalities could further extend synergistic harm reduction by building Radon mitigation into existing or new tobacco control initiatives (i.e., increasing cigarette taxes and spending the new revenue on Radon testing and mitigation support).

In 2013, Latz declared “Tobacco control policy is the most promising route to the public health goals of radon control policy”[51]. Since, the EPA, American Lung Association, and state of Kentucky have recently heeded the call[52–54]. As for the rest of us, the time to act is now. Because, over the next fifty years, we will be hard pressed to find any public health priority greater than eliminating the two main drivers of America’s deadliest cancer.

### Limitations

This study has several limitations. First, Radon exposure risk stratification may mask heterogeneity between counties within the high-risk group. We also, due to limited number of medium risk (2–4 pCi/L) counties, combined all counties not deemed high-risk. Second, Radon exposure was assessed at the county level, as was cancer incidence, which prohibited us from analyzing more granular geographic variation. Third, smoking data used as a control was limited to 2003–2011 and was not sex-stratified and did not account for intensity or duration. Fourth, the SEER-8 data includes four small states, which represent only 4% of the population. Application of our findings may only generalize to smaller and racially homogenous rural states. Finally, our analysis spanned a large time-period and did not attempt to adjust for dynamic policy landscapes related to smoking cessation or Radon mitigation. Finally, like all observational research, the design was subject to internal validity threats given the potential for unobservable county-level factors may be correlated with both the county-level Random Effect and Radon risk group. For this reason, we avoided causal language and reported our results in terms of estimated associations.

## Conclusions

As smoking rates in the United States continue to decline, public health systems balance prioritizing smoking cessation efforts with preventing secondary causes of lung cancer such as Radon. This study aimed to evaluate the relationship between high-risk residential radon exposure and lung cancer incidence by sex, histologic subtype, and decade over a nearly 50-year time period. Overall, we found that counties with high Radon accounted for most of the lung cancer cases and had a significantly higher age adjusted incidence rate when compared to low risk Radon counties. Most concerning, we found that the gap in lung cancer incidence between low and high-risk Radon counties were rising for females, resulting in a reversal of rankings for females in high-risk counties compared to males in low-risk counties. The limited regulatory efforts by the EPA have left the authority over protecting the public from the harms of Radon has fallen to states and municipalities, ultimately resulting in a vast landscape of radon testing and mitigation policies in both low and high-risk regions. Regardless of policy approaches, public health systems have an opportunity to capitalize growing interest in smoking cessation and Radon mitigation by creating novel programs which support both priorities in pursuit of reducing lung cancer risk for all Americans.

## Figures and Tables

**Table 1:** Summary Statistics

	Count	(%)	Person-Years	Age-Adjusted Incidence Rate
**Overall**	277,843	(100)	494,731,182	56.2
				
**Low Risk Counties**	86,090	(31.0)	208,761,159	45.4
**High Risk Counties**	191,753	(69.0)	285,970,023	60.0
				
**Connecticut**	115,584	(41.6)	162,472,669	63.9
**Iowa**	102,786	(37.0)	141,954,303	63.0
**New Mexico**	36,446	(13.1)	83,656,685	43.4
**Utah**	23,256	(8.4)	107,026,598	28.9
				
**1975–1984**	41,003	(14.8)	87,455,426	51.7
**1985–1994**	53,939	(19.4)	93,711,633	59.9
**1995–2004**	60,939	(21.9)	103,520,489	59.5
**2005–2014**	66,833	(24.1)	113,730,627	55.2
**2015–2022**	55,358	(19.9)	96,692,080	47.5
				
**Adenocarcinoma**	95,969	(34.5)	495,110,255	18.8
**Squamous cell carcinoma**	61,868	(22.3)	495,110,255	12.1
**Small cell carcinoma**	39,300	(14.1)	495,110,255	7.6
**Other**	80,935	(29.1)	495,110,255	16.0
				
**Female**	116,941	(42.1)	251,520,686	41.5
**Male**	161,131	(58.0)	243,589,569	71.8

Table 1 reports the summary statistics of lung cancer incidence case data from SEER-8 (1975–2022). Counties were classified as high-risk of Radon from EPA classification (> 4pCi/L)

**Table 2:** Results from Random Effects GLS Regression Testing for Differences in Lung Cancer Incidence by County-Level Radon Risk (1975–2022)

	Females and Males				
Coefficient	All Subtypes	Adenocarcinoma	Squamous Cell	Small cell	Other
High Risk County	13.5*** [10.0, 17.1]	5.2*** [4.0, 6.4]	4.3*** [3.1, 5.4]	2.7*** [−0.4, 1.7]	1.4* [0.0, 2.7]
					
High Risk X 1985–1994	7.3*** [3.7, 11.0]	3.5*** [1.8, 5.3]	1.5 [−0.2, 3.1]	2.3*** [1.2, 3.4]	0.1 [−1.7, 1.8]
High Risk X 1995–2004	8.4*** [4.7, 12.1]	5.0*** [3.0, 7.0]	0.1 [−1.5, 1.7]	2.1*** [0.9, 3.3]	1.2 [−0.6, 3.1]
High Risk X 2005–2014	11.5*** [7.8, 15.3]	7.7*** [5.5, 10.0]	0.9 [−0.5, 2.2]	2.7*** [1.5, 3.9]	0.2 [−1.6, 2.1]
High Risk X 2015–2022	13.1*** [9.0, 17.3]	7.5*** [5.6, 9.5]	1.9* [0.4, 3.4]	3.5*** [2.2, 4.7]	0.2 [−2.0, 2.4]
Wald Test Statistic (p)	0.014	< 0.001	0.063	0.019	0.545
					
	Females				
Coefficient	All Subtypes	Adenocarcinoma	Squamous Cell	Small cell	Other
High Risk County	6.8*** [3.9, 9.6]	3.6*** [2.4, 4.8]	1.6*** [0.8, 2.3]	1.7*** [1.1, 2.3]	−0.1 [−1.3, 0.4]
					
High Risk X 1985–1994	5.5** [2.0, 8.9]	2.9*** [1.3, 4.6]	1.3 [−0.7, 3.2]	1.5* [0.3, 2.7]	−.2 [−1.8, 1.4]
High Risk X 1995–2004	9.3*** [5.4, 13.1]	3.6** [1.2, 6.0]	1.4 [−0.2, 2.9]	1.9* [0.4, 3.5]	2.4 0.4, 4.3]
High Risk X 2005–2014	15.0*** [10.9, 19.0]	7.2*** [5.0, 9.5]	3.3** [2.3, 5.4]	3.1*** [1.9, 4.3]	1.3 [−0.8, 3.5]
High Risk X 2015–2022	18.5*** [14.5, 22.5]	8.1*** [5.6, 10.5]	3.9*** [1.8, 6.1]	4.5*** [3.3, 5.6]	2.1 [−0.8, 5.1]
*Wald Test Statistic (p)*	< *0.001*	< *0.001*	< *0.001*	< *0.001*	*0.025*
					
	Male				
Coefficient	All Subtypes	Adenocarcinoma	Squamous Cell	Small cell	Other
High Risk County	23.5*** [18.5, 28.4]	7.4*** [5.9, 8.9]	8.1*** [6.3, 9.9]	4.1*** [3.1, 5.1]	3.9*** [2.0, 5.8]
					
High Risk X 1985–1994	10.1** [3.9, 16.2]	4.4* [1.3, 7.5]	1.9 [−0.7, 4.6]	3.4** [1.4, 5.5]	0.3 [−2.9, 3.5]
High Risk X 1995–2004	6.4* [0.3, 12.4]	6.6*** [3.9, 9.3]	−2.0 [−4.9, 0.9]	2.0* [0.3, 3.8]	−.3 [−4.3, 1.6]
High Risk X 2005–2014	6.0 [−0.1, 12.2]	8.0*** [4.8, 11.1]	−2.6 [−5.7, 0.5]	2.1* [0.2, 3.9]	−1.4 [−4.3, 1.6]
High Risk X 2015–2022	4.6 [−2.4, 11.5]	6.7*** [3.7, 9.7]	−1.6 [−4.6, 1.5]	2.3* [0.2, 4.3]	−2.8 [−6.2, 0.6]
*Wald Test Statistic (p)*	*0.362*	*0.074*	*0.006*	*0.340*	*0.196*

Table 2 reports the results of the Generalized Least Squares (GLS) Random Effects regression model equations. All sex groups and histologic subtypes were estimated separately. The first row of each sex-specific result estimated the association between High-Risk Radon County (> 4 pCi/L) and lung cancer incidence rate. 95% confidence intervals reported in brackets. The second set of estimates, estimated simultaneously, interact the High-Risk County parameter with a decade-specific parameter, estimating the relative difference between low and high-risk counties in that decade compared to the difference in 1975–1984. The Wald Test Statistic (p-value) tests whether the estimated associations varied across each decade. With the Sidak method, Wald Test Statistic alpha significance level = 0.001. All rates were adjusted for age and reported as cases per 100,000 person-years.

* p <0.05,

** p <0.01,

*** p <0.001.

## Data Availability

The SEER-8 datafile used in this analysis is included as a supplemental file.

## Key Messages:

- Radon is the second leading cause of lung cancer, and the primary cause among non-smokers.
- High-risk Radon counties had higher incidence of lung cancer over a nearly 50-year period.
- In females only, the gap between low and high-risk Radon counties grew decade after decade.

## References

[1] NCI. Cancer of the Lung and Bronchus - Cancer Stat Facts [Internet]. SEER. [cited 2025 Aug 6]. Available from: https://seer.cancer.gov/statfacts/html/lungb.html

[2] Islami F, Goding Sauer A, Miller KD, Siegel RL, Fedewa SA, Jacobs EJ, Proportion and number of cancer cases and deaths attributable to potentially modifiable risk factors in the United States. CA: A Cancer Journal for Clinicians. 2018;68(1):31–54. 29160902 10.3322/caac.21440

[3] Jones J. Cigarette Smoking Rate in U.S. Ties 80-Year Low. Gallup [Internet]. 2024 Aug 13 [cited 2025 Aug 6]; Available from: https://news.gallup.com/poll/648521/cigarette-smoking-rate-ties-year-low.aspx

[4] Nkosi L. 20-Year Trends in Tobacco Sales and Self-Reported Tobacco Use in the United States, 2000–2020. Prev Chronic Dis [Internet]. 2022 [cited 2025 Aug 6];19. Available from: https://www.cdc.gov/pcd/issues/2022/21_0435.htm 10.5888/pcd19.210435 PMC9336190 35900882

[5] Jani CT, Singh H, Abdallah N, Mouchati C, Arora S, Kareff S, Trends in Lung Cancer Incidence and Mortality (1990–2019) in the United States: A Comprehensive Analysis of Gender and State-Level Disparities. JCO Glob Oncol. 2023 Sep;9:e2300255. 38127772 10.1200/GO.23.00255 PMC10752493

[6] Ward MM. Smoking Intensity and Mortality Across Age, Sex, and Race/Ethnicity Subgroups in the USA. J Racial and Ethnic Health Disparities [Internet]. 2025 Feb 27 [cited 2025 Aug 6]; Available from: 10.1007/s40615-025-02361-5 40016591

[7] Cunradi CB, Lee J, Pagano A, Caetano R, Alter HJ. Gender Differences in Smoking Among an Urban Emergency Department Sample. Tob Use Insights. 2019 Sep 26;12:1179173X19879136. 10.1177/1179173X19879136 PMC6763935 31598064

[8] Syamlal G, Mazurek JM, Dube SR. Gender Differences in Smoking Among U.S. Working Adults. Am J Prev Med. 2014 Oct;47(4):467–75. 25049215 10.1016/j.amepre.2014.06.013 PMC4542001

[9] Stone MD, Pierce JP, Dang B, McMenamin SB, Donaldson CD, Zhang X, State and Sociodemographic Trends in US Cigarette Smoking With Future Projections. JAMA Netw Open. 2025 Apr 25;8(4):e256834. 40279129 10.1001/jamanetworkopen.2025.6834 PMC12032569

[10] Eidy M, Regina AC, Tishkowski K. Radon Toxicity. In: StatPearls [Internet]. Treasure Island (FL): StatPearls Publishing; 2025 [cited 2025 Aug 6]. Available from: http://www.ncbi.nlm.nih.gov/books/NBK562321/32965992

[11] Lorenzo-González M, Torres-Durán M, Barbosa-Lorenzo R, Provencio-Pulla M, Barros-Dios JM, Ruano-Ravina A. Radon exposure: a major cause of lung cancer. Expert Rev Respir Med. 2019 Sep;13(9):839–50. 31318276 10.1080/17476348.2019.1645599

[12] Vogeltanz-Holm N, Schwartz GG. Radon and lung cancer: What does the public really know? Journal of Environmental Radioactivity. 2018 Dec 1;192:26–31. 29883874 10.1016/j.jenvrad.2018.05.017

[13] Bowie C, Bowie SH. Radon and health. Lancet. 1991 Feb 16;337(8738):409–13. 1671435 10.1016/0140-6736(91)91177-v

[14] Narayanan PK, Goodwin EH, Lehnert BE. Alpha particles initiate biological production of superoxide anions and hydrogen peroxide in human cells. Cancer Res. 1997 Sep 15;57(18):3963–71. 9307280

[15] Radiations NRC (US) C on the BE of I. Polonium. In: Health Risks of Radon and Other Internally Deposited Alpha-Emitters: Beir IV [Internet]. National Academies Press (US); 1988 [cited 2025 Aug 6]. Available from: https://www.ncbi.nlm.nih.gov/books/NBK218121/25032289

[16] IARC Working Group on the Evaluation of Carcinogenic Risks to Humans. RADON. In: Man-made Mineral Fibres and Radon [Internet]. Lyon (FR): International Agency for Research on Cancer; 1988 [cited 2025 Aug 6]. (IARC Monographs on the Evaluation of Carcinogenic Risks to Humans). Available from: https://www.ncbi.nlm.nih.gov/books/NBK316369/

[17] George AC. The history, development and the present status of the radon measurement programme in the United States of America. Radiat Prot Dosimetry. 2015 Nov;167(1–3):8–14. 25911413 10.1093/rpd/ncv213

[18] National Research Council (US) Committee on Evaluation of EPA Guidelines for Exposure to Naturally Occurring Radioactive Materials. Indoor-Radon Guidelines and Recommendations. In: Evaluation of Guidelines for Exposures to Technologically Enhanced Naturally Occurring Radioactive Materials [Internet]. National Academies Press (US); 1999 [cited 2025 Aug 6]. Available from: https://www.ncbi.nlm.nih.gov/books/NBK230646/25101423

[19] Field RW, Steck DJ, Smith BJ, Brus CP, Fisher EL, Neuberger JS, Residential radon gas exposure and lung cancer: the Iowa Radon Lung Cancer Study. Am J Epidemiol. 2000 Jun 1;151(11):1091–102. 10873134 10.1093/oxfordjournals.aje.a010153

[20] Reddy A, Conde C, Peterson C, Nugent K. Residential radon exposure and cancer. Oncol Rev. 2022 Mar 14;16(1):558. 35386751 10.4081/oncol.2022.558 PMC8977862

[21] Darby S, Hill D, Auvinen A, Barros-Dios JM, Baysson H, Bochicchio F, Radon in homes and risk of lung cancer: collaborative analysis of individual data from 13 European case-control studies. BMJ. 2005 Jan 29;330(7485):223. 15613366 10.1136/bmj.38308.477650.63 PMC546066

[22] Krewski D, Lubin JH, Zielinski JM, Alavanja M, Catalan VS, Field RW, Residential radon and risk of lung cancer: a combined analysis of 7 North American case-control studies. Epidemiology. 2005 Mar;16(2):137–45. 15703527 10.1097/01.ede.0000152522.80261.e3

[23] Ou JY, Fowler B, Ding Q, Kirchhoff AC, Pappas L, Boucher K, A statewide investigation of geographic lung cancer incidence patterns and radon exposure in a low-smoking population. BMC Cancer. 2018 Jan 31;18(1):115. 29385999 10.1186/s12885-018-4002-9 PMC5793382

[24] Lorenzo-González M, Ruano-Ravina A, Torres-Durán M, Kelsey KT, Provencio M, Parente-Lamelas I, Lung cancer and residential radon in never-smokers: A pooling study in the Northwest of Spain. Environmental Research. 2019 May 1;172:713–8. 30903971 10.1016/j.envres.2019.03.011

[25] Xiong Q, Zhang Z, Peng J, Liang J, Lian D, Zhao X, Epidemiological trends of lung cancer attributed to residential radon exposure at global, regional, and national level: a trend analysis study from 1990 to 2021. Front Public Health [Internet]. 2025 May 2 [cited 2025 Aug 6];13. Available from: https://www.frontiersin.org/journals/public-health/articles/10.3389/fpubh.2025.1593415/full 10.3389/fpubh.2025.1593415 PMC12081354 40385628

[26] Surveillance, Epidemiology, and End Results (SEER) Program (www.seer.cancer.gov) SEER*Stat Database: Incidence - SEER Research Data, 8 Registries, Nov 2024 Sub (1975–2022) - Linked To County Attributes - Time Dependent (1990–2023) Income/Rurality, 1969–2023 Counties, National Cancer Institute, DCCPS, Surveillance Research Program, released April 2025, based on the November 2024 submission.

[27] EPA. Map of Radon Zones [Internet]. 2014 [cited 2025 Aug 6]. Available from: https://www.epa.gov/radon/epa-map-radon-zones

[28] EPA O. What is EPA’s Action Level for Radon and What Does it Mean? [Internet]. 2019 [cited 2025 Aug 6]. Available from: https://www.epa.gov/radon/what-epas-action-level-radon-and-what-does-it-mean

[29] Mundlak Y. On the Pooling of Time Series and Cross Section Data. Econometrica. 1978;46(1):69–85.

[30] Schunck R. Within and between Estimates in Random-Effects Models: Advantages and Drawbacks of Correlated Random Effects and Hybrid Models. The Stata Journal: Promoting communications on statistics and Stata. 2013 Mar;13(1):65–76.

[31] Cabanillas OB, Michler JD, Michuda A, Tjernström E. Fitting and Interpreting Correlated Randomcoefficient Models Using Stata. The Stata Journal: Promoting communications on statistics and Stata. 2018 Mar;18(1):159–73.

[32] County Health Rankings. Adult Smoking [Internet]. 2011 [cited 2025 Aug 6]. Available from: https://www.countyhealthrankings.org/health-data/iowa

[33] Hamiye Beyaztas B, Bandyopadhyay S. Robust estimation for linear panel data models. Stat Med. 2020 Dec 20;39(29):4421–38. 32901960 10.1002/sim.8732

[34] Bottai M, Orsini N. Confidence Intervals for the Variance Component of Random-effects Linear Models. The Stata Journal: Promoting communications on statistics and Stata. 2004 Dec;4(4):429–35.

[35] Šidák Z. Rectangular Confidence Regions for the Means of Multivariate Normal Distributions. Journal of the American Statistical Association. 1967 Jun 1;62(318):626–33.

[36] Alavanja MC, Lubin JH, Mahaffey JA, Brownson RC. Residential radon exposure and risk of lung cancer in Missouri. Am J Public Health. 1999 Jul;89(7):1042–8. 10394313 10.2105/ajph.89.7.1042 PMC1508843

[37] CDC. Lung Cancer Among People Who Never Smoked [Internet]. Lung Cancer. 2025 [cited 2025 Aug 6]. Available from: https://www.cdc.gov/lung-cancer/nonsmokers/index.html

[38] Glass Geltman EA. State Radon Laws [Internet]. The Policy Surveillance Program. 2016 [cited 2025 Aug 6]. Available from: https://legacy.lawatlas.org/datasets/state-radon-laws

[39] Environmental Law Institute. Database of State Indoor Air Quality Laws [Internet]. 2025 [cited 2025 Aug 6]. Available from: https://www.eli.org/buildings/database-state-indoor-air-quality-laws

[40] Ford ES, Kelly AE, Teutsch SM, Thacker SB, Garbe PL. Radon and lung cancer: a cost-effectiveness analysis. Am J Public Health. 1999 Mar;89(3):351–7. 10076484 10.2105/ajph.89.3.351 PMC1508623

[41] Puskin JS, Nelson CB. EPA’s perspective on risks from residential radon exposure. JAPCA. 1989 Jul;39(7):915–20. 2769311 10.1080/08940630.1989.10466577

[42] Meenakshi C, Mohankumar MN. Synergistic effect of radon in blood cells of smokers - an in vitro study. Mutat Res. 2013 Sep 18;757(1):79–82. 23850733 10.1016/j.mrgentox.2013.06.018

[43] CDC. Current Cigarette Smoking Among Adults in the United States [Internet]. Smoking and Tobacco Use. 2024 [cited 2025 Aug 6]. Available from: https://www.cdc.gov/tobacco/php/data-statistics/adult-datacigarettes/index.html

[44] Lee ME, Lichtenstein E, Andrews JA, Glasgow RE, Hampson SE. Radon-smoking synergy: A populationbased behavioral risk reduction approach. Prev Med. 1999 Sep;29(3):222–7. 10479611 10.1006/pmed.1999.0531

[45] Lichtenstein E, Andrews JA, Lee ME, Glasgow RE, Hampson SE. Using radon risk to motivate smoking reduction: evaluation of written materials and brief telephone counselling. Tob Control. 2000 Sep;9(3):320–6. 10982577 10.1136/tc.9.3.320 PMC1748368

[46] Lichtenstein E, Boles SM, Lee ME, Hampson SE, Glasgow RE, Fellows J. Using radon risk to motivate smoking reduction II: randomized evaluation of brief telephone counseling and a targeted video. Health Educ Res. 2008 Apr;23(2):191–201. 17426046 10.1093/her/cym016

[47] Hampson SE, Andrews JA, Barckley M, Lichtenstein E, Lee ME. Personality traits, perceived risk, and risk-reduction behaviors: a further study of smoking and radon. Health Psychol. 2006 Jul;25(4):530–6. 16846328 10.1037/0278-6133.25.4.530 PMC2409280

[48] Kim S, Chiu T, Klug MG, Schmitz D, Schwartz GG. Interventions to promote home radon testing: A randomized clinical trial of a smartphone app vs. printed brochures. Cancer Med. 2023 Jan;12(2):2027–32. 35762397 10.1002/cam4.4988 PMC9883538

[49] Schmitz D, Klug MG, Schwartz GG. Short Communication: Radon testing via a state tobacco quitline. Prev Med Rep. 2024 Jun;42:102738. 38689887 10.1016/j.pmedr.2024.102738 PMC11059320

[50] Smyth R, Long S, Wiseman E, Sharpe D, Breen D, O’Regan A. Radon testing in rapid access lung clinics: an opportunity for secondary prevention. Ir J Med Sci. 2017 May;186(2):485–7. 27083463 10.1007/s11845-016-1448-0

[51] Lantz PM, Mendez D, Philbert MA. Radon, Smoking, and Lung Cancer: The Need to Refocus Radon Control Policy. Am J Public Health. 2013 Mar;103(3):443–7. 23327258 10.2105/AJPH.2012.300926 PMC3673501

[52] EPA. The National Radon Action Plan - A Strategy for Saving Lives [Internet]. 2015 [cited 2025 Aug 6]. Available from: https://www.epa.gov/radon/national-radon-action-plan-strategy-saving-lives

[53] ALA AL. Smoking Combined with Radon Exposure Significantly Increases Risk for Lung Cancer [Internet]. 2024 [cited 2025 Aug 6]. Available from: https://www.lung.org/media/press-releases/fy25-radonsmoking-news-release

[54] Huntington-Moskos L, Rayens MK, Wiggins A, Hahn EJ. Radon, Secondhand Smoke, and Children in the Home: Creating a Teachable Moment for Lung Cancer Prevention. Public Health Nurs. 2016 Nov;33(6):529–38. 27443982 10.1111/phn.12283 PMC5118164

